# Enhancing faba bean (*Vicia faba* L.) productivity through establishing the area-specific fertilizer rate recommendation in southwest Ethiopia

**DOI:** 10.1515/biol-2022-0844

**Published:** 2024-04-02

**Authors:** Berhanu Bilate Daemo

**Affiliations:** Department of Plant Science, Wolaita Sodo University, Dawro Tarcha Campus, P.O. Box 138, Tarcha, Ethiopia

**Keywords:** faba bean, grain yield, NPSB rate, soil fertility management

## Abstract

The aim of this study is to establish area-specific NPSB (18.9% N, 37.7% P_2_O_5_, 6.95% S, and 0.1% B) fertilizer rate recommendations for the optimal grain yield of faba bean. The field experiment was conducted in two locations in the 2021 and 2022 cropping seasons using a randomized complete block design with three replications. The nine treatments included 0, 25, 50, 75, 100, 125, 150, 175, and 200 kg ha^−1^ NPSB fertilizer rates. An economic analysis was conducted for grain yield using the International Maize and Wheat Improvement Center procedure. The analysis of variance results showed that blended fertilizer significantly (*p* < 0.01) affected plant height, number of pods per plant, number of seeds per plant, hundred seeds weight, biomass yield, grain yield (GY), and harvest index. The combined location mean result showed that applying a 125 kg ha^−1^ NPSB rate produced the highest GY (4857.2 kg ha^−1^). The result of economic analysis demonstrated that applying a 125 kg ha^−1^ NPSB rate earned the highest net benefits (212824.0 ETB ha^−1^) and marginal rate of return (3653.43%). Therefore, a 125 kg ha^−1^ NPSB fertilizer rate is recommended for high yield and profitability of faba bean production in the study area and other similar soil types.

## Graphical abstract

**Figure:**
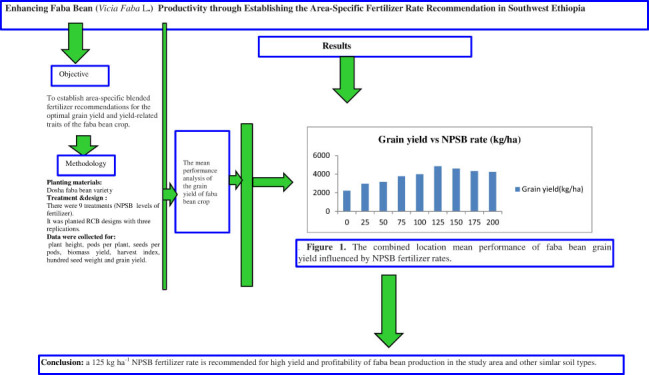

## 1 Introduction

Faba bean (*Vicia faba* L.) is rich in protein; it can reach 25.4%, and is ranked second to soybean in protein content [1,2,3]. In the world, it has been cultivated in over 60 different countries, and China is the leading producer of faba beans, followed by Ethiopia, Egypt, and the United Kingdom [1,4,5]. Among pulse crops, in terms of cultivated area and production, the faba bean ranks third behind the soybean and the field pea in the world [6]. Ethiopia accounts for 56% of the total faba bean production in Africa, and it is the most significant source of protein in the diet of the most smallholder farmers in the region [1,4]. In the 2020/2021 cropping season, about 4.5 million smallholder farmers produced 1078265.5 metric ton in total production from an area of 518466.39 ha [7]. However, Ethiopia frequently confronts a severe and ongoing food crisis as a result of its low supply of agricultural production, which is characterized by low productivity per unit area and ineffective agricultural practices [2,3,8]. For example, the present estimated average yield of faba bean on smallholder farmer farms in Ethiopia is 2.1 t ha^−1^ [7], which is significantly lower than the global average yield of 4.8 t ha^−1^ [9]. Low yields in Ethiopia are caused by poor soil fertility management, as well as other variables such as soil erosion, poor weeding practices, and the presence of various pests and diseases [4,10].

In tropical Africa, maintaining soil fertility is a serious problem, particularly in countries with rapid population growth like Ethiopia [11,12,13]. Because declining soil fertility is a key issue for faba bean production, an adequate and balanced supply of nutrients is required for optimal faba bean yield [13,14,15]. It is a fact that inorganic fertilizers have proven to be an effective tool for managing soil fertility problems, which have contributed significantly in enhancing food production. Proper utilization of chemical fertilizer can dramatically maximize yield and turn otherwise unproductive soil into productive soil [16,17]. Chemical fertilizers are estimated to contribute one-third to one-half of total agricultural production, and thus, they are an indispensable agricultural input [18]. It is expected that an increase in crop productivity of 40–60% is thought to be attributable to the use of commercial fertilizers [19]. Because of this, increased agricultural production without a balanced application of fertilizers has resulted in soil fertility exhaustion and nutrient imbalance in plants, including problems with macro- and micronutrients [10,20]. Therefore, to optimize crop yield and meet the food needs of families with ever-expanding population growth, growing improved varieties along with chemical fertilizer may need to become an integral part of the farming operation in developing countries [21,22].

According to Ethiopian soil analysis data, the country’s soils were significantly low in most nutrients, including nitrogen (86%), phosphorus (99%), sulfur (92%), boron (65%), zinc (53%), and potassium (7%) [23,24,25]. Due to this, blended multi-nutrient fertilizers have been developed to address area-specific nutrient deficits and thus boost crop production and productivity. Farmers in the study area are currently using a newly released blended NPSB fertilizer with a blanket recommendation of 100 kg ha ^−1^, which contains nutrients (18.9% N, 37.7% P_2_O_5_, 6.95% S, and 0.1% B) [23,25]. However, the blanket recommended (100 kg ha^−1^ NPSB) fertilizer rate was generated based on other soil types elsewhere, and so far, this has not been demonstrated in the study area for the optimum dose for faba bean production. As a result, it is challenging for smallholder farmers and scholars to comprehend the appropriate levels of NPSB fertilizers for faba bean production. Hence, crop yield can be increased more economically and sustainably by better matching fertilizer use to local climate, soil, and management practices [10,25,26,27]. Additionally, Bekele et al. [25] suggested shifting from blanket fertilizer recommendations toward site-specific nutrient management that is based on a thorough understanding of the differences in crop response with respect to soil type, agro-ecology, and suitable soil and plant studies. This indicated that there was a need to develop area-specific NPSB fertilizer rate recommendations to enhance the productivity and production of faba bean for smallholder farmers. Therefore, the study aimed to establish area specific blended fertilizer rate for better faba bean grain yield (GY) and yield related traits.

To achieve this objective, the study conducted soil analysis at the trial sites for two seasons before planting and arranged the nine NPSB fertilizer rates based on 100 kg ha^−1^ NPSB rate fertilizer as a basis, which is the rate farmers currently apply for faba bean production. Additionally, it used a randomized complete block design to control the two source variations of landscape, and the lottery randomization method was used to assign treatment to each plot. Finally, the research standard procedure involved collecting data from randomly selected individual plants and the net plot area of the whole plant.

## 2 Materials and methods

### 2.1 Description of the study area

The field experiment was conducted under rain-fed conduction in the 2021 and 2022 main cropping seasons at the Waka and Kachi trial sites. The detailed description of study areas is presented in Table 1.

**Table 1:** Geographical location, climate, and soil type of trial sites

Description	Waka	Kachi	Reference
Altitude (m a.s.l)	2,450	2,090	[28,29]
Latitude	7^o^03′07″N	7^o^1′4.11″N	[28,29]
Longitude	37^o^11′37″E	37^o^0’26.56″E	[28,29]
Annual rainfall (mm)	1,550–2,605	1,443–2,535	[28,29]
Temperature (min–max)	9.24–20.85°C	11.7–23.5°C	[28,29]
Soil type	Alisols	Luvisols	[28,29]

### 2.2 Soil sampling and analysis

Before planting, surface (0–30 cm depth) soil samples were obtained using an auger from ten randomly selected points across the experimental fields of the Waka and Kachi trial sites and composited into one sample. A 1.0 kg sample was obtained from this mixture, air-dried, crushed, and sieved through a 2 mm sieve. The soil texture was determined using the Bouyoucos hydrometer [30]. The soil pH was measured potentiometrically with a digital pH meter in the supernatant suspension of a 1:2:5 soil-to-water ratio [31]. Total nitrogen was determined using the Kjeldahl method by Bremner [32]. Exchangeable calcium (Ca), potassium (K), magnesium (Mg), sulfur (S), phosphorus (P), and boron (B) were determined by the procedures described by Mehlich [33]. The cation exchange capacity (CEC) was determined using the ammonium acetate method by Chapman [34]. The details of the testing site soil results are presented in Table 2.

**Table 2:** Selected physico-chemical properties of soils of experimental sites in 2021 and 2022 cropping season

Soil properties	Unit	Environment		Rating	Reference
		Waka	Kachi		
Sand	%	28	20		
Clay	%	30	38		
Silt	%	42	42		
Textural class		Clay loam	Clay		
pH	—	4.66	5.29	Strong acid (5.1–5.5)	[35]
Total nitrogen	%	0.20	0.24	Medium (0.15–0.25)	[35]
Phosphorous	mg/kg	4.73	2.75	very low avail. (<15)	[35]
Sulfur	mg/kg	9.71	8.25	Very low (<10)	[35]
Calcium	mg/kg	2312.09	2212.11	High (2,000–4,000)	[35]
Magnesium	mg/kg	206.71	210.12	Moderate (120–360)	[35]
Potassium	mg/kg	269.34	275.35	Optimum (190–600)	[35]
CEC	cmol(+)/kg soil	37.48	37.73	High (25–40)	[35]
Boron	mg/kg	0.48	0.02	Deficiency (<0.5)	[35]

### 2.3 Experimental material, treatment, design of experiment, and field management

As a test crop, the faba bean variety Dosha was used. Holata Agricultural Research Centre developed the Dosha variety in 2009, and it is a well-adapted variety in the study area. The farmer’s utilization rate (100 kg ha^−1^ NPSB) was used as the basis for arranging the treatment. The treatments included nine NPSB fertilizer levels (0, 25, 50, 75, 100, 125, 150, 175, and 200 kg ha^−1^). At planting time, the whole NPSB fertilizer was administered at the rate specified per plot. The trial sites had two sources of variation (slope and soil fertility), and hence, to control this variation, the study used a randomized complete block design with three replications. Each treatment was assigned to each plot using the lottery randomization method, and then each fertilizer rate was distributed uniformly to each plot. The experimental plot was 4 m in width and 3 m in length, with 0.5 m and 1 m between the experimental unit and block, respectively. The land was ploughed three times before planting, and the seed of the faba bean was sown at a spacing of 0.4 m and 0.1 m between the row and plant, respectively. A starter nitrogen fertilizer was applied at a rate of 18 kg ha^−1^ in the form of urea during the planting period. Weeding was done two times (35 and 55 days after planting) uniformly. Diseases and pests were monitored and protected until the crops were harvested from the field.

### 2.4 Data collection

#### 2.4.1 Plant height (PH; cm)

In the middle rows of each plot, ten randomly selected plants were measured from the soil surface to the tip of the stem at 90% physiological maturity.

#### 2.4.2 Number of pods per plant (NPP) and the number of seeds per pod (NSP)

The NPP and NSP were counted from the central rows of ten randomly selected plants per plot at harvest and their mean was used for analysis.

#### 2.4.3 Aboveground dry biomass yield (BY; kg ha^−1^)

At 90% physiological maturity, ten randomly selected plants per plot from the middle rows were measured after 7 days of sun drying in the field until constant weight and converted from kg per plot to kg per hectare for analysis.

#### 2.4.4 Grain yield (GY; kg ha^−1^)

From the net plot area (9.6 m^2^), faba bean was harvested, threshed, cleaned, and weighed. Also, the GY was corrected to a moisture content of 10% using a moisture tester. Then, its value (kg per plot) was converted to kg per hectare for analysis.

#### 2.4.5 Hundred seeds weight (HSW; g)

Hundred seed weight was sampled from cleaned seeds of each plot and counted using an electronic counter. Then, it was measured using a sensitive balance by correcting the moisture content of the seed to 10%.

#### 2.4.6 Harvest index (HI; %)

HI was calculated as follows:

(1) \[\text{HI}=\text{Weight of grain yield}/(\text{Weight of aboveground dry biomass yield})\text{}\times \text{}100.]\]

### 2.5 Agronomic data and economic analysis

SAS statistical software, version 9.4 [36], was used to perform the data analysis of variance, and the mean separation was done using the least significant difference (LSD) at a 5% level of significance. Each location and season agronomic data were subjected to analysis variance (ANOVA), and the normality test was performed using the Shapiro–Wilk W test. Also, an individual location and season data homogeneity test was performed using Bartlett’s test, and then the combined analysis of variances was performed after confirming the homogeneity of the error variances. The combined ANOVA was performed using Generalized linear model procedures for randomized complete block design [37] as follows:

(2) \[{Y}_{{ixjk}}=\mu +{T}_{i}+{S}_{x}+{L}_{j}+{{TS}}_{{ix}}+{{TL}}_{{ij}}+{{TSL}}_{{ixj}}+{R}_{k}+{{\mathrm{\varepsilon }}}_{{ixjk}},]\]

where \[{Y}_{{ixjk}}\hspace{1em}]\] is the observed value of trait \[i]\] in replication \[k]\] of season *x* and location \[j]\] , *μ* is the grand mean of the trait, \[{T}_{i}]\] is the effect of trait *i*, \[{S}_{x}]\] is the effect of season *x*, \[{l}_{j}]\] is the effect of location *j*, \[{{TS}}_{{ix}}]\] is the interaction effect of trait \[i]\] with season *x*, \[{{TL}}_{{ij}}]\] is the interaction effect of trait \[i]\] with location *j*, \[{{TSL}}_{{ixj}}]\] is the interaction effect of trait \[i]\] with season *x* and location *j*, \[{R}_{k}]\] is the effect of replication \[k]\] , and \[{{\mathrm{\varepsilon }}}_{{ixjk}}]\] is the error (residual) effect of trait \[i]\] in replication *k* of season *x* and location \[j]\] .

A partial budget analysis was calculated for each treatment to consolidate the statistical analysis of the agronomic data. According to the International Maize and Wheat Improvement Center (CIMMYT) procedure [32], farmers would achieve yields 10% lower than the obtained yield in the experiment, and then the mean faba bean GY was adjusted in the economic analysis by subtracting 10% from the actual yield. Economic evaluations were computed for the total variable cost (TVS), gross field benefit, net benefit (NB), and marginal rate of return (MRR) ratios using the method described by CIMMYT [38].

### 2.6 TVC

The TVC was determined as the sum of all variable costs (the cost of chemical fertilizer and labor costs for the application of the fertilizer), and the other costs remained constant for each treatment. The cost of NPSB fertilizer was 42.20 ETB kg^−1^ and the cost of application of NPSB fertilizer was 500.00 ETB ha^−1^. (3)

### 2.7 Gross field benefits (GFBs)

GFB was obtained by multiplying the adjusted total GY (kg ha^−1^) for each treatment by the current open market price of kg per Ethiopian birr (50.00 ETB kg^−1^) for the faba bean. (4)

### 2.8 Net benefit (NB)

NB was obtained as the difference between the GFB and the TVC. (5)

### 2.9 MRR (%)

The MRR was computed using the formula: \[{\mathrm{MRR}}( \% )=\frac{\triangle {\mathrm{NB}}}{\triangle {\mathrm{TVC}}}\times 100,]\] where \[\triangle {\mathrm{NB}}]\] was the change in NB and \[\triangle {\mathrm{TVC}}]\] was the change in TVC between any pair of treatments. (6)

## 3 Results and discussion

### 3.1 PH

Different levels of NPSB fertilizer application had a significant (*p* < 0.001) effect on the PH of the faba bean (Table 3). The tallest PH of 109.7 cm at Waka and 107.3 cm at the Kachi were recorded by applying a 150 kg ha^−1^ NPSB blended fertilizer rate (Table 4). In both locations, the unfertilized plot had the smallest PH (Table 4). Also, the combined location mean revealed that applying a 150 kg ha^−1^ NPSB blended fertilizer rate resulted in the highest PH (108.5 cm), followed by 105.3 cm by applying a 175 kg ha^−1^ NPSB fertilizer rate (Table 4). The PH of faba bean increased dramatically as the NPSB fertilizer rate increased from 0 to 150 kg ha^−1^. The study hypothesized that the optimal availability of N, P, S, and B fertilizers could promote vegetative growth and result in increased PH in the faba bean. This study, like Genetu et al. [4] in faba bean and Tadesse et al. [39] in common bean, found that applying inorganic fertilizer significantly boosted the PH.

**Table 3:** Combined analysis of variance of locations over seasons for growth, yield, and yield related traits of faba bean grown at Waka and Kachi in 2021 and 2022

Source of variations	DF	Mean squares						
		PH	NPP	NSP	HSW	BY	GY	HI
Treatment (T)	8	1012.7^**^	21.68^**^	3.18^**^	601.18^**^	15614269.9^**^	9385440.94^**^	485.68^**^
Location (L)	1	197.3^*^	3.34^NS^	0.45^NS^	948.14^**^	374768.9^*^	2427300.75^**^	396.75^**^
Year (Y)	1	1841.8^**^	15.56^*^	0.01^NS^	186.7^*^	13962.8^NS^	762888.23^*^	118.23^*^
Replication	2	205.7^NS^	7.44^NS^	0.45^NS^	6.37^NS^	7155388.1^NS^	231109.36 ^NS^	28.58^NS^
T × L	8	8.49^NS^	1.23^NS^	0.03^NS^	7.96^NS^	10551.6^NS^	214716.40^NS^	31.27^NS^
T × Y	8	13.68^NS^	0.46^NS^	0.46^NS^	38.05^NS^	15383.9^NS^	50366.38^NS^	5.58^NS^
T × Y × L	8	126.25^NS^	3.43^NS^	0.08^NS^	16.05^NS^	82989.6^NS^	215991.88^*^	28.84^NS^
Residual	70	37.23	1.89	0.35	42.94	938591.9	99492.35	23.85

Key: NS, *, **, = non-significant at 0.05, significant at 0.05, and highly significant 0.01 level of probability, respectively, PH = plant height (cm), NPP = number of pods per plant, NSP = number of seeds per pod, HSW = hundred seeds weight (g), BY = biomass yield (kg ha^−1^), GY = grain yield (kg ha^−1^), HI = harvest index (%).

**Table 4:** NPSB fertilizer application influenced the PH (cm), NPP, and NSP of faba bean grown at Waka and Kachi in 2021 and 2022

NPSB rate (kg ha^−1^)	PH (cm)			NPP			NSP		
	Waka	Kachi	Mean value	Waka	Kachi	Mean value	Waka	Kachi	Mean value
0	83.0^d^	75.8^e^	79.4^f^	8.3^c^	6.8^d^	7.6^d^	2.5^c^	2.7^cd^	2.6^e^
25	87.0^d^	85.2^d^	86.1^c^	10.2^b^	9.7^c^	9.9^c^	2.3^c^	2.3^d^	2.3^d^
50	100.5^bc^	98.5^bc^	99.5^cd^	10.3^b^	9.8^c^	10.1^c^	2.5^c^	2.5^d^	2.5^d^
75	96.8^c^	94.8^c^	95.8^d^	10.2^b^	9.7^c^	9.9^c^	2.7^bc^	2.8^bcd^	2.8^cd^
100	100.6^bc^	98.5^bc^	99.6^cd^	10.8^ab^	10.5^bc^	10.6^bc^	3.0^bc^	3.3^abc^	3.2^bc^
125	101.5^bc^	104.2^ab^	102.9^bcn^	12.3^a^	11.8^ab^	12.1^a^	3.8^a^	3.6^a^	3.7^a^
150	109.7^a^	107.3^a^	108.5^a^	11.3^ab^	11.7^ab^	11.5^ab^	3.7^a^	3.8^a^	3.8^a^
175	106.5^ab^	99.3^bc^	102.9^bc^	11.3^ab^	12.2^a^	11.8^ab^	3.3^ab^	3.5^ab^	3.4^ab^
200	102.6^abc^	100.3^bc^	101.5^bc^	10.5^b^	10.0^c^	10.3^c^	3.3^ab^	3.3^abc^	3.3^ab^
LSD (5%)	7.7	6.7	4.9	1.6	1.6	1.1	0.7	0.7	0.5
CV	7.9	9.4	8.9.	13.3	13.4	13.2	19.52	18.7	18.6

Mean values within the same column followed by the same letter or no letters are not significantly different.

### 3.2 NPP and NSP

The NPP and NSP were significantly influenced by applying an NPSB fertilizer (Table 3). Applying a 125 kg ha^−1^ NPSB fertilizer rate resulted in the highest NPP (12.3) at the Waka, whereas applying a 175 kg ha^−1^ NPSB fertilizer rate resulted in the highest NPP (12.2) at the Kachi (Table 4). The combined location mean result showed that the highest NPP (12.1) were produced by the application of 125 kg ha^−1^ NPSB fertilizer rate, followed by 11.8 pods per plant by the application of 175 kg ha^−1^ NPSB fertilizer rate, which was statistically similar (Table 4). The result demonstrated that raising the NPSB fertilizer rate from 0 to 125 kg ha^−1^ would significantly increase the NPP, while increasing the NPSB fertilizer rate from 125 to 200 kg ha^−1^, a varied value was observed (Table 4). The improved availability of nutrients in the soil solution for plants following the optimal application of NPSB fertilizer may be the cause of the increased NPP.

At the Waka, the highest NSP (3.8) were produced by applying 125 kg ha^−1^ NPSB fertilizer rate, followed by 3.7 seeds per plant by applying a 150 kg ha^−1^ NPSB rate, while at the Kachi, the highest NSP (3.8) were produced by the application of the 150 kg ha^−1^ NPSB fertilizer rate (Table 4). The combined location mean performance result showed that the highest seeds per pod (3.8) were produced by applying a 150 kg ha^−1^ NPSB fertilizer rate, followed by 3.7 seeds per pod by applying a 125 kg ha^−1^ NPSB fertilizer rate (Table 4). The study indicates that there is a linear relationship between the NPP and the NSP with NPSB fertilizer application. Hence, the optimum rate of blended fertilizer application can significantly increase the NPP and the NSP in faba beans. The highest NPP and NSP in faba bean were observed in the earlier studies by Nebiyu et al. [40] and Rasul [41] with an application rate of 150 and 125 kg ha^−1^ NPS fertilizer, respectively. A similar study by Zamukulu et al. [42] reported that NPS fertilizer significantly affected the NPP and the NSP in common beans.

### 3.3 HSW

The HSW was significantly affected by the application of NPSB fertilizer rates (Table 3). At the Waka, applying a 150 kg ha^−1^ NPSB fertilizer rate resulted in the highest HSW (104.2 g), followed by 103.5 g and 102.7 g by the application of 125 and 175 kg ha^−1^ NPSB fertilizer rates, respectively, which were statistically similar, while the lowest HSW (85.3 g) was found in an unfertilized plot (Table 5). Similarly, at Kachi, the highest HSW (98.3 g) was obtained by applying a 125 kg ha^−1^ NPSB fertilizer rate (Table 5). The pooled location mean result revealed that the highest HSW (100.9 g) was produced by applying a 125 kg ha^−1^ NPSB fertilizer rate (Table 5). This finding indicates that increasing the fertilizer rate from 0 to 125 kg ha^−1^ would increase (82–100.9 g) the HSW of the faba bean; beyond that, it shows no increment in the HSW. The observed difference in HSW could be attributed to an increase in the rate of P, which will help balance the utilization of other minerals. This finding is corroborated by earlier research by Mekonnen and Saliha [43], Deresa [44], and Zewide et al. [45], who found a considerable increase in common bean HSW following the application of blended fertilizer.

**Table 5:** NPSB fertilizer application influenced the HSW (g) and HI (%) of faba bean grown at Waka and Kachi in 2021 and 2022

NPSB rate (kg ha^−1^)	HSW (g)			HI (%)		
	Waka	Kachi	Mean value	Waka	Kachi	Mean value
0	85.3^d^	78.7^d^	82.0^e^	30.5^c^	28.0^e^	29.3^e^
25	85.6^d^	83.8^cd^	84.7^e^	45.0^ab^	34.8^d^	39.8^b^
50	90.0^cd^	84.2^cd^	87.1^de^	34.8^c^	37.5^cd^	36.2^d^
75	93.6^bc^	87.5^bc^	90.6^cd^	42.8^b^	37.8^cd^	40.4^b^
100	99.0^ab^	92.0^ab^	95.5^bc^	43.5^ab^	40.2^abc^	43.8^bc^
125	103.5^a^	98.3^a^	100.9^a^	51.3^a^	45.8^a^	48.6^a^
150	104.2^a^	96.8^a^	100.5^ab^	47.0^ab^	43.5^ab^	45.3^ab^
175	102.7^a^	96.3^a^	99.5^ab^	43.0^b^	38.3^bcd^	40.6^bc^
200	98.3^ab^	91.3^abc^	94.8^bc^	45.8^ab^	41.3^bcd^	43.6^bc^
LSD (5%)	7.9	7.6	5.3	6.4	5.6	4.2
CV	9.8	7.8	8.9	13.0	17.47	12.8

Mean values within the same column followed by the same letter or no letters are not significantly different.

### 3.4 HI

Applying an NPSB fertilizer significantly influenced the HI trait in the faba bean (Table 3). Applying a 125 kg ha^−1^ NPSB rate gave the maximum HI (51.3%) and (45.6%) at the Waka and Kachi locations, respectively (Table 5). The unfertilized plot produced the lowest harvest index (30.5%) at the Waka and (28.0%) at the Kachi (Table 5). Additionally, analysis of the combined location mean separation showed that the highest HI (48.6%) was achieved by applying a 125 kg ha^−1^ NPSB rate, followed by 45.3 and 43.8% produced by the application of 150 and 100 kg ha^−1^ NPSB fertilizer rates, respectively (Table 5). This study suggests that an increase in the blended NPSB fertilizer rate leads to an increase in HI. This could be the positive effect of NPSB fertilizer on crop nutrient utilization and photoassimilate movement from vegetative to grain sections of the crop. This conclusion is consistent with Gebeyehu’s [46] observation that the application of NPSB to faba bean caused the HI to significantly increase. Furthermore, Deresa [44] reported that NPS fertilizer application had a considerable effect on the common bean HI, and Debela et al. [47] observed strong NPS effects on the soybean HI.

### 3.5 BY

**Figure 1:**
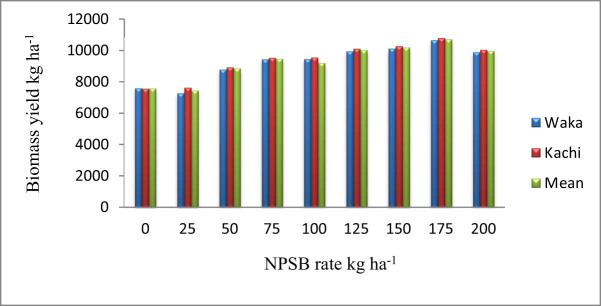
The NPSB fertilizer application influenced the aboveground dry BY (kg ha^−1^) of faba bean grown at Waka and Kachi in 2021 and 2022.

The application of various levels of NPSB blended fertilizer had a substantial effect on the aboveground dry BY (Table 3). At the Waka, the application of 175 kg ha^−1^ NPSB rate resulted in the highest dry BY (10,609 kg ha^−1^), followed by 10,093 kg ha^−1^ by applying 150 kg ha^−1^ NPSB rate, and the lowest dry BY (7245.7 kg ha^−1^) was recorded by the application of 25 kg ha^−1^ NPSB blended fertilizer rate (Figure 1). Similarly, at Kachi, applying 175 kg ha^−1^ NPSB fertilizer rate gave the highest dry BY (10,743 kg ha^−1^), while the lowest dry BY (7545.3 kg ha^−1^) was produced from an unfertilized plot (Figure 1). Furthermore, analysis of the combined location mean result indicated that the highest dry biomass yield (10,676 kg ha^−1^) was produced by applying a 175 kg ha^−1^ NPSB rate (Figure 1). The results showed that higher NPSB fertilizer rates were related to higher biomass yields. According to this finding, increasing blended NPSB rates leads to enhanced availability of nitrogen, which could increase vegetative growth and raise the yield of aboveground dry biomass. This result is similar to the study by Samago et al. [48], who found that NP fertilizers significantly increased dry BY and dry matter production in common beans, and Tirfessa et al. [49] found NPSB application significantly enhanced common bean BY.

### 3.6 GY

**Figure 2:**
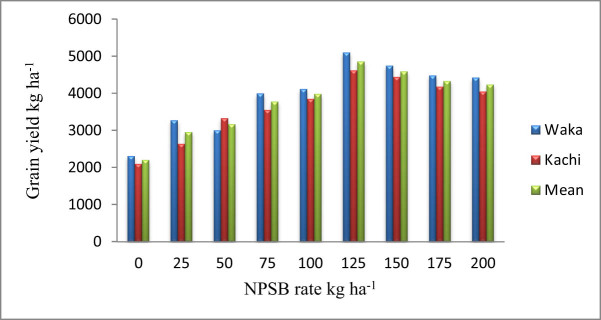
The NPSB fertilizer application influenced the GY (kg ha^−1^) of faba bean grown at Waka and Kachi in 2021 and 2022.

Applying various doses of NPSB blended fertilizer considerably influenced the GY of faba beans (Table 3). Applying a 125 kg ha^−1^ NPSB rate resulted in the highest GYs (5098.8 kg ha^−1^) and (4615.5 8 kg ha^−1^) at the Waka and Kachi, respectively (Figure 2). The unfertilized plots produced the lowest GY (2311.3 kg ha^−1^) at the Waka and (2099.0 kg ha^−1^) at the Kachi. As a result, at the Waka, the highest GY was exceeded by 120.64 and 23.88% unfertilized plots and blanket recommended fertilizer produced GY, respectively, while at the Kachi, the maximum grain yield was greater than 119.5 and 20.0% unfertilized plots and blanket recommended fertilizer produced GY, respectively. Additionally, the combined location mean performance result showed that the highest grain yield (4857.2 kg ha^−1^) was produced by the application of 125 kg ha^−1^ NPSB rate, followed by 4589.8 kg ha^−1^ GY by the application of 150 kg ha^−1^ NPSB rate (Figure 2). The findings show that increasing the NPSB fertilizer rate from 0 to 125 kg ha^−1^ would increase (from 2206.9 to 4857.2 kg ha^−1^) the GY of the faba bean, beyond which increasing the fertilizer rate would gradually reduce the GY. This implies that a 125 kg ha^−1^ NPSB fertilizer rate could be the maximum requirement for the faba bean in the current study. The increased GY in faba beans is due to higher S and B fertilizers, their availability accompanied by major nutrients and enhanced crop uptake. This leads to improved chlorophyll absorption and dry matter production, which contribute to the GY increase. The study observed the presence of a linear relationship between GY and NPSB blended fertilizer application. Thus, smallholder farmers can boost faba bean GY using the optimum rate of NPSB fertilizer.

In an earlier study, Gebeyehu [46] found the highest GY with the application of 150 kg ha^−1^ NPSB, which increased GY by 154% compared to the unfertilized plot, and Geleta and Bekele [50] reported that the application of 120 NPSB produced the maximum GY in faba bean, where it exceeded that of the unfertilized plot GY by 178%. Similar findings that different rates of blended fertilizer significantly influenced GY were reported by Kaysha et al. [51] in mung bean; Baza et al. [52] in mung bean; Arega and Zenebe [53] in common bean; Debela et al. [47] in soybean.

### 3.7 Soil acidity

The soil analysis results (Table 2) shows that the pH of the trial sites had a low value ranging from 4.66 to 5.29, and a soil pH less than 5.5 could influence crop yield due to the toxicities of H^+^, Al^3+^, Mn^2+^, and Fe^2+^ cations, as well as the concomitant in the applied phosphorus (P) could not be fully available to crops [54]. In this case, liming based on soil test findings is crucial to raising the pH of the soil up to the desired level advised for crops [55]. As a result, the study expected that if the trial sites were limed, the linear response of the examined traits to NPSB application would be greater than the observed value of the current finding.

### 3.8 Economic analysis

The partial budget analysis result is presented in Table 6. The partial budget analysis was determined using the TVCs and the NBs of each treatment, and it was calculated based on the combined location GY mean value. The cost of NPSB fertilizer and the labor associated with applying it varied in the current study, whereas all other expenditures remained the same for each treatment. In terms of GY and NBs, applying NPSB blended fertilizer to faba bean crops was often superior to that of unfertilized plots. As a result, the present study findings showed that the highest NB (212824.0 ETB ha^−1^) along with the highest MRR (3653.43%) was recorded by applying a 125 kg ha^−1^ NPSB blended fertilizer rate, followed by NBs of 174463.0 ETB ha^−1^ with 788% of the MRR by the application of 100 kg ha^−1^ NPSB blended fertilizer rate (Table 6). The findings indicate that a 125 kg ha^−1^ blended fertilizer rate is a superior option for smallholder farmers to increase the productivity and production of faba beans in the study area and other similar agro-ecologies and soil types.

**Table 6:** Partial budget analysis for NPSB fertilizer rate based on combined location mean GY of faba bean grown at Waka and Kachi in the 2021 and 2022 cropping seasons

NPSB (kg ha^−1^)	UGY (kg ha^−1^)	AGY (kg ha^−1^)	Fertilizer cost (ETB ha^−1^)	Fert. app. cost (ETB ha^−1^)	TVC (ETB ha^−1^)	GFB (ETB ha^−1^)	NB (ETB ha^−1^)	MRR
0	2206.9	1986.21	0	0	0	99310.5	99310.5	
25	2955	2659.5	1050.0	500.0	1550.0	132975.0	131425.0	2071.9
50	3167	2850.3	2100.0	500.0	2600.0	142515.0	139915.0	808.57
75	3774.2	3396.78	3150.0	500.0	3650.0	169839.0	166189.0	2502.29
100	3981.4	3583.26	4200.0	500.0	4700.0	179163.0	174463.0	788.0
125	4857.2	4371.48	5250.0	500.0	5750.0	218574.0	212824.0	3653.43
150	4589.8	4130.82	6300.0	500.0	6800.0	206541.0	199741.0	D
175	4327.5	3894.75	7350.0	500.0	7850.0	194737.5	186887.5	D
200	4234.5	3811.05	8400.0	500.0	8900.0	190552.5	181652.5	D

Notice: 1US Dollar = 55 ETB current exchanging rate. Key: UGY = unadjusted grain yield kg ha^−1^, AGY = 10% adjusted gain yield kg ha^−1^, TVC = total variables costs (ETB), GFB = gross field benefit (ETB), NB = net benefit (ETB), MRR = marginal rate of return (%), and D = dominated.

## 4 Conclusion

The use of chemical fertilizers is the most common agricultural practice for increasing crop yield. However, their utilization beyond or below optimal levels has resulted in negative effects on the environment and crop yield. Hence, chemical fertilizer rate recommendations need to be improved for economic and environmental reasons. As a result, the present study was conducted to determine the NPSB blended fertilizer rate for the optimal GY and other agronomic traits of the faba bean. The results demonstrated that NPSB fertilizer application had a substantial effect on PH, NPP, NSP, HSW, aboveground BY, GY, and HI traits of the faba bean. The optimal fertilizer rate for faba bean production is 125 kg ha^−1^ NPSB, resulting in the highest mean GY (4857.2 kg ha^−1^), NBs (212824.0 ETB ha^−1^), and MRR (3653.43%). Therefore, a 125 kg ha^−1^NPSB fertilizer rate is suggested for high yield and profitability of faba bean production in the study area and other similar soil types and agro-ecological zone. The finding could be applied to both acidic and non-acidic soil.

### 4.1 Limitation of the study

Our findings present the NPSB fertilizer application effect on faba bean GY and yield-related traits research method based on the stated objective. In this study, soil fertility management mainly focused on blended chemical fertilizer to improve faba bean GY and yield-related traits. However, the soil test result indicated that the trial sites had a high acidity level, which required lime treatment. Hence, liming is believed to be able to raise the soil pH optimum level, increase the availability of phosphorus (P), and minimize the toxicities of H^+^, Al3^+^, Mn^2+^, and Fe^2+^ cations, resulting in a positive effect on crop yield. Therefore, the author is encouraged to take the variable lime into account with NPSB fertilizer in future studies.
